# Asymmetric Properties of the Heart Rate Microstructure in Healthy Adults during 48 h ECG Recordings

**DOI:** 10.3390/jcm12237472

**Published:** 2023-12-02

**Authors:** Greta Sibrecht, Jarosław Piskorski, Tomasz Krauze, Przemysław Guzik

**Affiliations:** 1 Department of Cardiology–Intensive Therapy, Poznan University of Medical Sciences, Przybyszewskiego 49, 60-355 Poznan, Poland; 2 Institute of Physics, University of Zielona Gora, Szafrana 4a, 65-516 Zielona Gora, Poland; jaropis@zg.home.pl

**Keywords:** HRA, HR microstructure, sex differences, 48 h Holter ECG

## Abstract

Heart rate asymmetry reflects the different contributions of heart rate (HR) decelerations and accelerations to heart rate variability (HRV). We examined the contribution of monotonic runs of HR accelerations and decelerations to the asymmetric properties of the HR microstructure in the 48 h electrocardiograms (ECGs) of healthy adults (n = 101, 47 males, average age of 39 years) and analysed sex differences in the HR microstructure. The HR microstructure was asymmetric for runs of most lengths, except for sequences of two consecutive decelerations (DR2s) or accelerations (AR2s). Women had a higher prevalence of AR2s than men but fewer runs in the range of 4 to 11 consecutive accelerations (AR4–AR11s) and 5 to 11 consecutive decelerations (DR5–DR11s). The longest runs consisted of 47 consecutive accelerations (AR47s) and 27 consecutive decelerations (DR27s). More DR3s than AR3s and more DR4s than AR4s reveal a crossing of HR microstructure asymmetry. In conclusion, more acceleration than deceleration runs demonstrate that the HR microstructure was asymmetric in the 48 h ECGs. This phenomenon was present in both sexes but was more pronounced in men. For shorter runs of 3 and 4 consecutive heartbeats, there was a crossing of HR microstructure asymmetry, with more deceleration than acceleration runs.

## 1. Introduction

Heart rate variability (HRV) measures the beat-to-beat changes in the duration of the cardiac cycles of sinus origin. In an ECG, cardiac cycles are represented by the distances between successive QRS complex peaks and are called RR intervals. These intervals are usually used to calculate HRV [1,2].

Many internal and external factors influence the electrical activation of the sinus node and the propagation of electrical depolarisation to other cardiac cells [3,4,5,6,7,8]. Internal factors include direct mechanical effects on the heart, like breathing [9] or changes in the amount of blood returning to both atria [10]. Some other examples are the balance of sympathetic and parasympathetic activity [11], body temperature changes [12], electrolytes, hypoxia [13,14], and hormones and cytokines [15]. Daylight, weather conditions, temperature and humidity, environmental pollution and noise are external factors modifying instantaneous heart rate (HR) [16,17,18]. The sinus node is a complex and multicellular structure. Therefore, it is unlikely that only two cells can generate an action potential of the same length during two consecutive cardiac cycles. The process of initiating and regulating HR is complex and multifactorial. This explains why the interpretation of HRV is also complex and context dependent.

Many mathematical algorithms can be applied to analyse the duration of cardiac cycles or RR intervals to quantify HRV. Guzik and Piskorski, using Poincaré plots of RR intervals, discovered and described heart rate asymmetry (HRA) [19,20,21,22]. This phenomenon is caused by unequal contributions of HR accelerations and decelerations to various features of HRV, such as variance, structure, complexity and trends. Specifically, HR decelerations contribute more to short-term HRV [19] but less to long-term and total HRV [20]. HRA has been studied in ECG recordings with lengths ranging from 1 min to 24 h [19,20,21,22]. We have recently shown that variance-based HRA was present in the 48 h ECGs of healthy adults and that HRA expression was stronger in women than men [23].

Monotonic runs refer to a consecutive sequence of values that either increase or decrease over time in a time series analysis. RR intervals, an example of a time series, can be grouped into continuously increasing or decreasing series of monotonic runs. A series of consecutive RR interval prolongations represent HR deceleration runs (DRs), while a series of RR interval shortenings represent HR acceleration runs (ARs). These runs may have different lengths determined by the number of heartbeats changing in the same direction (increasing or decreasing). Some examples are a single deceleration (DR1), a pair of accelerations (AR2s), a run of five consecutive decelerations (DR5s) or nine accelerations (AR9s).

HR monotonic runs form the HR microstructure and have asymmetric features [24]. HR decelerations usually contribute less than HR accelerations to the HR microstructure. Piskorski and Guzik studied 87 Holter ECG recordings from healthy subjects, showing that the number of different-length ARs was significantly higher than that of DRs. Impaired asymmetric features of the HR microstructure have been reported in survivors of acute myocardial infarction at an increased risk of mortality [25], premature infants at risk of sepsis [26], patients with severe obstructive sleep apnoea [27,28], chronic obstructive pulmonary disease [29], type 2 diabetes mellitus and hypertension [30].

Asymmetrical features of HR microstructure were studied using ECG recordings between 30 min and 24 h. The distribution of DRs and ARs, and the length of the longest DRs and ARs, depended on the duration of the ECGs. The longest runs are usually up to 8 consecutive HR decelerations or accelerations in ECGs of a 30 min duration, whereas they can be up to 16–18 beats in length in 24 h ECGs. No similar study has explored the HR microstructure in ECGs lasting over 48 h. Additionally, no study has investigated sex differences in the HR microstructure.

Another issue in determining the type and number of monotonic runs is the sampling frequency of the recorded ECGs. In our previous study of 24 h ECGs, the sampling frequency was 200 Hz, with 6–7% of all RR intervals being neutral runs created by consecutive RR intervals of identical duration. Due to the complex structure and physiology of the sinus node, the probability of two consecutive cardiac cycles having the same duration is close to 0%, not 6–7%. We have suggested that such a higher proportion of neutral runs is caused by technical issues and a low sampling frequency rather than the physiological properties of the sinus HR [24].

The aim of this study was to investigate the presence of asymmetric features of the HR microstructure using 48 h ECGs acquired at a sampling frequency of 8000 Hz from healthy adults. It also investigated sex differences in HR microstructure asymmetry. Finally, we analysed the proportion of neutral runs in such long ECGs.

## 2. Materials and Methods

### 2.1. Participants

In total, 116 adults aged between 19 and 60 years old were enrolled in this study. The inclusion criteria were voluntary participation, signing informed consent, and self-reported good health. The exclusion criteria included any chronic disease or acute illness within the past three months, or professional athletes [23]. A Holter-ECG recordings in which the total number of technical artefacts or non-sinus beats exceeded 10% of the recording was also excluded. Finally, the data of 101 participants were used for further analysis. Holter ECG recordings were stored on a computer using Medilog Darwin 2 Enterprise (Schiller, Baar, Switzerland) software and then transferred to an external hard drive. The data were collected and managed using REDCap electronic data capture tools hosted at the Poznan University of Medical Sciences [31,32].

### 2.2. 48 h ECGs

The participants underwent a three-channel ECG Holter recording conducted using the Medilog AR12plus device (Schiller, Baar, Switzerland). The recordings were captured at a sampling frequency of 8000 Hz, and the recording lasted up to 48 h with a minimum duration of 36 h. The recordings were automatically analysed and reviewed manually to ensure all beats were correctly classified. The duration of each RR interval and information about its origin, such as sinus, supraventricular, ventricular, or technical artefacts, were exported to MATLAB to calculate the HRV and HRA. Publicly available, open-source software (HRAexplorer.com) was used to calculate the HRA parameters with only RR intervals of sinus origin and duration within the 300–1800 ms range used to compute the HRV and HRA.

### 2.3. Heart Rate Microstructure Measurement

Methodological details on the HR microstructure and its asymmetry have been described elsewhere, with accompanying graphs [33]. Shortly, the RR intervals were divided into monotonic runs of different lengths to analyse the HR microstructure, as already explained. Each RR interval (${RR}_{i+1}$) was compared with the previous RR interval (${RR}_{i}$). Any ${RR}_{i+1}$ > ${RR}_{i}$ corresponded to HR deceleration, whereas any ${RR}_{i+1}$ < ${RR}_{i}$ corresponded to HR acceleration. For each ${RR}_{i+1}$ = ${RR}_{i}$, there was no change in HR.

Runs with no change in HR on successive beats were considered neutral. In this way, all sinus RR intervals were divided into groups of deceleration runs (DRs), acceleration runs (ARs) and neutral runs (NRs), all with a wide range of length from 1 to tens of RR intervals.

Counting statistics were then used to quantify the DRs, ARs and NRs. For each run of a different length, the number of all beats producing such a run was divided by the number of all sinus beats in the following way:

DRn = N_DRn_/N_all_ [%] for deceleration runs,

ARn = N_ARn_/N_all_ [%] for acceleration runs,

NRn = N_NRn_/N_all_ [%] for neutral runs,

where N_DRn_ is the number of sinus beats generating deceleration runs of length n, N_ARn_ is the number of sinus beats generating acceleration runs of length n, N_NRn_ is the number of sinus beats generating neutral runs of length n and N_all_ is the number of all sinus beats. In this way, the relative contribution of each type of monotonic run of a given length to all sinus beats was quantified [24,25,33].

We limited our analysis to monotonic runs of 25 consecutive and concordant HR decelerations or HR accelerations. For neutral runs, only those consisting of 1, 2 or 3 consecutive identical RR intervals were examined, as longer neutral runs of >3 consecutive RR intervals were virtually absent.

### 2.4. Statistical Analysis

Continuous data distribution was graphically analysed using histograms and Q–Q plots, and the d’Agostino–Pearson test was applied if necessary [34]. As most data had a non-Gaussian distribution, they were summarised using the median and the 25th (Q1) and 75th (Q3) percentiles. The Mann–Whitney (M-W) *U* test was applied to compare men and women. Accelerating and decelerating series of equal length were compared using the paired Wilcoxon test. The Fisher exact test was used for categorical comparisons. A *p*-value < 0.05 was considered statistically significant. Statistical analyses were performed using PQStat software (PQStat v.1.8.4.138, PQStat Software, Poznan, Poland) and Jamovi (version 2.3; www.jamovi.org).

## 3. Results

### 3.1. Clinical Characteristics

Table 1 presents the clinical characteristics of the included subjects, and, of note, 16 were current smokers and 19 were ex-smokers. There were no statistically significant differences between males and females.

### 3.2. The HR Microstructure during 48 h ECG Recordings

Table 2 shows the results of the HR microstructure analysis for all participants. The rates of acceleration runs were significantly higher (except for DR2 vs. AR2, DR3 vs. AR3 and DR4 vs. AR4) than deceleration runs of the same lengths, with asymmetry present in all runs except for length 2 (DR2 vs. AR2).

### 3.3. Sex Differences in the HR Microstructure during 48 h ECG Recordings

Table 3 presents the HR microstructure analysis results according to sex, showing that the HR microstructure is asymmetric in women except for the runs of length 2 (DR2 vs. AR2). The number of acceleration runs was significantly higher (except for DR2 vs. AR2, DR3 vs. AR3 and DR4 vs. AR4) than deceleration runs, with no differences between men and women. The HR microstructure is asymmetric in men for all the runs longer than 2. There were some sex differences, with women having more AR2s and men having more of the following deceleration runs: DR5—DR11 and DR17, and acceleration runs: AR4–AR11 and AR13.

### 3.4. Crossing of the Acceleration and Deceleration Run Contributions

**Figure 1:**
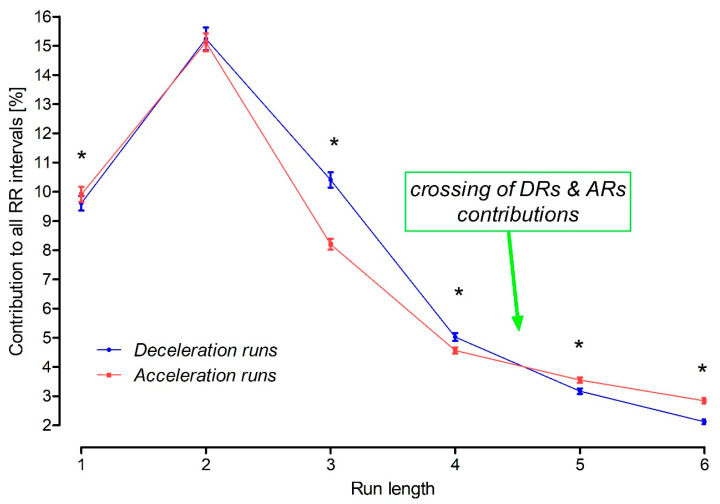
Summary of the contribution of RR intervals during acceleration and deceleration runs to the total RR intervals of sinus origin. Data are presented as mean and standard errors of the mean for presentation purposes. Significantly more AR1s than DR1s are present, with no significant difference between AR2s and DR2s. Conversely, there were more DR3s and DR4s than AR3s and AR4s. Acceleration runs were more frequent than deceleration runs of the same length, starting from runs of length 5 (See Table 2 for detailed results and Table 3 for separate analyses for men and women). *—a significant difference with *p* at least <0.05 or less by the Wilcoxon test for paired data.

There were generally more ARs than DRs, except runs of two, three and four consecutive beats. The AR2 and DR2 rates were also comparable in all subjects, separately in men and women. However, there was reverse dominance for AR3 vs. DR3 and AR4 vs. DR4, with more decelerations than accelerations for runs of lengths 3 or 4. This phenomenon was present in all the subjects studied and was present in men and women independently (Figure 1). In addition, there were no sex differences for DR3s, DR4s and AR3s.

### 3.5. The Longest Runs

The longest runs comprised 47 consecutive accelerations (AR47s) and 27 decelerations (DR27). The median values for the longest ARs and DRs were 26 (23–30) and 16 (15–18), respectively, and these differences were significant. In women, the average longest run was formed by 26 accelerations (AR26s, 22–29) and 16 decelerations (DR16s, 15–19), and similarly in men, by 26 accelerations (AR26s, 23–30) and 16 decelerations (DR16s, 15–18). There were no differences between the groups.

### 3.6. Neutral Runs

The observed neutral runs contributed to less than 1% of all beats in the whole group (Table 4). The contributions of NR1, NR2 and NR3 compared with corresponding AR1 and DR1 or AR2 and DR2 or AR3 and DR3 were always significantly lower (*p* < 0.000001).

## 4. Discussion

This study revealed that healthy individuals have an asymmetric HR microstructure in their 48 h ECGs. This asymmetry is present in both sexes, but men have lower AR2 and higher AR4 values than women, and more acceleration and deceleration runs from 5 to 11 (AR5–AR11 and DR5–DR11). Moreover, the rate of neutral runs is almost negligible in these recordings, likely due to the high ECG sampling frequency.

### 4.1. HRA in Men and Women

Sex differences in HRV have been extensively studied and systematically analysed. A meta-analysis [35] showed that HRV is more pronounced in men than women, but it was limited by including recordings with durations ranging from 10 cardiac cycles to 24 h, mixing children with adults, and including athletes and non-athletes. It has also been reported that adult men have a stronger expression of short-term, long-term and total HRA than women in 30 min recordings [36]. We have demonstrated that HRA has a greater expression in men than women using 48 h ECGs and variance-based measures. Specifically, SD1d, SD2d, SD2a, SDNNd, SDNNa, C1d and CLa were higher in men, while C2d, CTd, Nd and CLd were lower in men than women. However, the prevalence of all forms of HRA and its compensation mechanisms appear to be similar between the sexes [23].

Previous studies have not examined sex differences in HR microstructural asymmetries. We demonstrate that the HR microstructure is asymmetric in both men and women, with notable sex differences. Women showed a higher prevalence of AR2, whereas men showed a higher frequency of longer strands, particularly DR5–DR11 and DR17, and acceleration strands AR4–AR11 and AR13. In a similar way to the variance-based HRA, the asymmetric microstructural features of the HR are more strongly expressed in healthy men than in women.

### 4.2. The HR Microstructure in Healthy Individuals

Studies on the HR microstructure and its asymmetric features were initiated by our group in 2011 [24] in healthy people. We showed that the rate of ARs of different lengths was significantly higher than that of DRs using 24 h Holter ECG recordings sampled at 200 Hz. Runs of lengths 3 and 4 behaved differently. DR3 and DR4 appeared more numerous (but not significantly) than AR3 and AR4. By contrast, the number of DR3s and DR4s were significantly higher than AR3s and AR4s in this investigation, possibly due to the increased statistical power for the AR3 vs. DR3 and AR4 vs. DR4 comparisons. This may be caused by the twice-as-long ECGs and 40-times-higher sampling signal frequency.

### 4.3. Clinical Value of the HR Microstructure

The largest clinical study on the HR microstructure [25] was performed on survivors of acute myocardial infarction who underwent 24 h ECGs during hospitalisation. Patients with increased DR1 and reduced DR2–DR10 were at higher mortality risk in the 2-year follow-up. Among patients with DR4 <0.05%, all-cause mortality rates were 24% in the training group (1455 patients) and 21.9% in the validation group (946 patients). Cardiac death rates were 17.3% and 17.2%, respectively, while sudden cardiac death rates were 6.7% and 9.4%.

The predictive value of single accelerations and decelerations was investigated in pre-exercise ECGs of at least 1 min duration obtained from 944 consecutive patients in the Finnish Cardiovascular Study [37]. All patients underwent treadmill exercise tests and were followed for a mean of almost 57 months. People with an increased AR1 (>16.85%) or DR1 (>17.7%) had a significantly higher all-cause mortality risk of cardiovascular death and sudden cardiac death than other patients. There was an essential similarity with the study in post-infarction patients [25]: high-risk patients in these two groups had increased AR1s and DR1s.

The HR microstructure has also been studied in preterm infants [26]—those at higher risk of sepsis had fewer DR1s and more DR3s and DR4s. More decelerations forming longer runs were observed at the expense of fewer DR1s.

Patients with severe obstructive sleep apnoea syndrome (OSAS) had fewer DR1s and AR1s than patients with moderate OSAS and more AR5s, AR10s, DR5s and DR8s than subjects with no or mild OSAS [27]. Jiang et al. [28] confirmed that OSAS patients had longer HR acceleration and deceleration runs, also showing that the HR microstructure improved in OSAS patients after treatment with continuous positive pressure applied overnight. Both DR4 and DR8 were associated with a higher risk of supraventricular arrhythmias, and DR8 alone better predicted ventricular arrhythmias in 24 h Holter ECGs. DRs may be an indicator of arrhythmia risk in COPD patients [29].

Wang et al. analysed 24 h Holter ECGs of subjects with type 2 diabetes (DM2) with/without hypertension, showing that the DR2s, DR4s and DR8s in patients with DM2 (with or without hypertension) were lower than in healthy subjects [30]. This study also suggested that HbA1c (glycated haemoglobin) might be the most important contributor to the change in DRs, and that insulin resistance was negatively correlated with the rate of DRs.

### 4.4. Physiological Crossing of the Asymmetric Features of the HR Microstructure

HRV arises from changes in the duration of RR intervals, i.e., HR accelerating and decelerating. Neutral RR intervals either do not have or have a random contribution depending on HRV characteristics and sampling frequency. Different scenarios for such HR acceleration/deceleration distributions are possible. For instance, HR accelerations and decelerations may contribute similarly, randomly or differently to the count, variance and structure-based HRV features. However, physiologically, there is one dominant scenario known as HRA: HR accelerations and decelerations consistently contribute unequally to HRV. HR decelerations have a greater contribution to short-term HRV but lesser contribution to long-term HRV for the variance-based characteristics of HRA. This is the HRA compensation phenomenon [24].

Monotonic runs of different lengths that form the HR microstructure also intersect and interchange. There are more HR accelerations than decelerations for runs of the length 1, 2 and over 4. Previously, no difference was found in the rates of AR3 vs. DR3 and AR4 vs. DR4 in ECGs recorded at a lower sampling frequency of 200 Hz. In this current study, the ECG signal was recorded at a sampling frequency of 8000 Hz, and more deceleration runs were observed than acceleration runs of 3 and 4 consecutive beats. Similar to our previous reports, there were more AR1s than DR1s, as well as longer runs of 5, i.e., AR5s and so on, than deceleration runs of the same length. AR2 and DR2 rates were not significantly different. The double-crossing phenomenon of the rate of the runs between 1 and >4 has not been observed. This newly described crossing phenomenon is probably another feature of HRA compensation, but its potential mechanisms remain unknown.

The instantaneous HR is coupled to the respiratory cycle [38]—HR increases during inspiration and decreases during expiration [39]. Due to respiratory sinus arrhythmia, HR changes from acceleration to deceleration several times per minute, every minute for 24 h. Respiratory sinus arrhythmia can be affected by sleep state [40] or momentary physical activity [41], with the average person taking 10–15 breaths at rest. Inspiration usually lasts 1–1.5 s and expiration lasts 1.5–2 s. With each expiration, the heart slows down for 3–4 beats, producing most of the DR3s and DR4s. Thus, the crossing phenomenon observed in our study group might be related to expiration [42].

### 4.5. Sampling Frequency

In the previous study of the HR microstructure using 24 h ECGs sampled at a frequency of 200 Hz [24], the number of neutral runs was up to 6–7% of all beats. Guzik and Piskorski also showed that a lower sampling rate (down to 100 Hz) underestimates HR run-based entropy due to an artificial increase in more neutral runs. In clinical studies on the HR microstructure, neutral runs were not reported [25,26,28,29,30,37].

Using the sampling frequency of 8000 Hz reduced the number of neutral runs to less than 1%. Physiologically, no consecutive two beats of the heart are the same [2], and therefore, it remains a question of which sampling frequency is needed to provide a complete picture of the HR microstructure. It may be technically impossible to measure the duration of each cardiac cycle in the ECG so precisely that no two consecutive RR intervals are identical. Nevertheless, information on sampling frequency and the number of neutral runs should be reported in all studies of the HR microstructure.

### 4.6. Study Limitations

This study involved adults aged between 19 and 60 years; therefore, the results cannot be extrapolated to those over 60 or to children. Also, the participants were healthy; therefore, the results may vary in patients with different illnesses. The volunteers were from the Polish population, i.e., mainly of European ethnicity, therefore, the findings cannot be generalised to other ethnic groups. None of the participants were taking any medication; therefore, we cannot derive any information about pharmacological agents; however, our data can be used as a reference for other studies investigating these issues.

### 4.7. Novelty, Potential Clinical Meanings and Conclusions

This is the first study to demonstrate asymmetric features of the HR microstructure in healthy individuals during long-term 48 h ECGs. We also show for the first time that asymmetric features of the HR microstructure are present in both men and women. Furthermore, there are sex differences in the rate of shorter and middle runs, mainly between 5 and 11 consecutive beats, but not for the longest runs.

In addition, the exceptionally high ECG sampling frequency of 8000 Hz is crucial in discriminating more accurately between heart rate accelerations and decelerations. Observing the crossing of deceleration and acceleration runs became possible by reversing their rates between AR4 and DR4 and AR5 and DR5. In addition, the high ECG sampling frequency significantly reduces the proportion of consecutive RR intervals of identical duration. Consequently, the reported neutral run rates are very low, reflecting theoretical and physiological considerations. Further studies are required to explain this finding.

Our findings can be used as a reference for other physiological, methodological and clinical studies. ECG durations of up to 48 h and higher sampling frequencies help to better identify longer ARs and DRs. Furthermore, very long ECG recordings of up to several weeks are becoming the new standard with modern Holter systems, ECG biopatches or implanted cardiac devices. Indeed, such monitoring is possible for up to 2 years with implantable loop recorders. Wearable devices using photoplethysmography can also monitor heart rate over longer periods, measured in weeks, months and years. These technologies are more commonly used to diagnose atrial fibrillation, atrial flutter and other arrhythmias, but whether the analysis of the asymmetric features of the heart rate microstructure will have additional clinical, diagnostic or predictive value is to be seen.

### 4.8. Conclusions

Overall, this study shows that the HR microstructure in 48 h ECGs from healthy adults exhibits asymmetry characterised by a higher prevalence of HR accelerations than HR decelerations, and HR accelerations also produce much longer monotonic runs than HR decelerations. There is a crossing of HR microstructure asymmetry for monotonic runs of length 3 and 4 consecutive RR intervals with more DR3s than AR3s and more DR4s than AR4s. In addition, the HR microstructure is asymmetric in both men and women, with significantly greater expression in men. Finally, the number of neutral runs consisting of consecutive RR intervals of equal duration decreases when the ECG is recorded at a sampling frequency of 8000 Hz.

## Figures and Tables

**Table 1:** Participants’ clinical characteristics.

Parameter (Median, IQR)	All Participants (n = 101)	Women (n = 54)	Men (n = 47)
Age [years]	39 (28–44)	40 (28–45)	39 (28–43)
BMI [kg/m²]	23.7 (21–25.6)	22.5 (20.8–25)	24.5 (22–26.8)
SBP [mmHg]	118 (114–124)	118 (114–124)	117 (114–124)
DBP [mmHg]	72 (67–79)	75 (68–80)	71 (66–78)
HR [beats/min]	70 (65–75)	70 (65–75)	70 (63–75)

Abbreviations: IQR—interquartile range; BMI—body mass index; SBP—systolic blood pressure; DBP—diastolic blood pressure; HR—heart rate.

**Table 2:** Comparison of the relative contribution of deceleration and acceleration runs of different lengths to the total number of sinus beats derived from 48 h ECG recordings of healthy individuals (n = 101).

Length of Run	Acceleration Runs [%]		Deceleration Runs [%]		*p*-Value (Wilcoxon Test)
	Median	IQR	Median	IQR	
1	9.80	7.87–11.41	9.22	7.99–10.88	0.046
2	14.70	12.52–17.20	14.63	12.59–18.16	0.750
3	8.17	6.80–9.67	10.48	8.64–12.06	<0.001
4	4.42	3.76–5.32	4.97	4.06–5.78	<0.001
5	3.52	2.92–4.12	3.12	2.52–3.94	<0.001
6	2.72	2.24–3.41	2.14	1.52–2.67	<0.001
7	2.14	1.62–2.66	1.24	0.85–1.60	<0.001
8	1.54	1.05–1.89	0.64	0.45–0.90	<0.001
9	0.98	1.67–1.32	0.31	0.21–0.50	<0.001
10	0.60	0.40–0.93	0.16	0.10–0.26	<0.001
11	0.42	0.27–0.60	0.08	0.05–0.14	<0.001
12	0.25	0.17–0.42	0.04	0.02–0.08	<0.001
13	0.20	0.11–0.28	0.03	0.01–0.05	<0.001
14	0.14	0.07–0.22	0.01	0–0.03	<0.001
15	0.10	0.05–0.15	0.01	0–0.02	<0.001
16	0.06	0.04–0.10	0	0–0.01	<0.001
17	0.05	0.2–0.08	0	0–0.01	<0.001
18	0.03	0.01–0.07	0	0–0	<0.001
19	0.03	0.01–0.05	0	0–0	<0.001
20	0.02	0.01–0.04	0	0–0	<0.001
21	0.01	0–0.03	0	0–0	<0.001
22	0.01	0–0.02	0	0–0	<0.001
23	0	0–0.01	0	0–0	<0.001
24	0	0–0.01	0	0–0	<0.001
25	0	0–0.01	0	0–0	<0.001

Abbreviations: IQR—interquartile range.

**Table 3:** Comparison of the relative contributions of deceleration and acceleration runs of different lengths to the total number of sinus beats for men and women and the analysis of sex differences within the HR microstructure derived from 48 h ECG recordings of healthy individuals.

Run Length	Women ^					Men ^					* *p*-Value (M-W)	# *p*-Value (M-W)
	Acceleration Runs * [%]		Deceleration Runs # [%]		^ *p*-Value (Wilcoxon) Test)	Acceleration Runs * [%]		Deceleration Runs # [%]		^ *p*-Value (Wilcoxon)		
	Median	IQR	Median	IQR		Median	IQR	Median	IQR			
1	9.88	8.76–11.26	9.30	8.29–10.62	0.025	9.33	7.68–11.42	9.01	7.76–11.06	0.694	0.5040	0.8191
2	16.37	13.88–18.03	16.28	13.90–18.56	0.718	12.94	11.89–15.38	13.30	11.10–15.63	0.320	0.0001	0.1009
3	8.25	7.37–9.85	10.64	9.32–12.55	<0.001	8.06	6.52–9.10	9.45	7.77–11.26	<0.001	0.1978	0.4830
4	4.00	3.45–4.66	4.40	3.88–5.53	<0.001	5.00	4.12–5.61	5.46	4.46–5.88	<0.001	0.0004	0.6620
5	3.23	2.77–3.62	2.77	2.15–3.19	<0.001	3.89	3.22–4.72	3.89	3.02–4.37	<0.001	0.0001	0.0012
6	2.47	2.09–2.97	1.86	1.25–2.27	<0.001	3.21	2.71–3.95	2.56	2.06–2.90	<0.001	0.0001	0.0397
7	1.79	1.45–2.27	1.10	0.69–1.47	<0.001	2.50	2.08–2.93	1.38	1.13–1.69	<0.001	0.0001	0.0017
8	1.21	0.91–1.74	0.57	0.29–0.85	<0.001	1.71	1.29–2.12	0.72	0.52–0.98	<0.001	0.0016	0.0001
9	0.780	0.60–1.17	0.29	0.16–0.48	<0.001	1.10	0.77–1.43	0.33	0.24–0.53	<0.001	0.0063	0.0001
10	0.53	0.36–0.84	0.15	0.08–0.24	<0.001	0.77	0.50–1.01	0.20	0.12–0.28	<0.001	0.0119	0.0042
11	0.38	0.22–0.54	0.08	0.05–0.13	<0.001	0.51	0.30–0.66	0.09	0.05–0.14	<0.001	0.0189	0.0231
12	0.24	0.16–0.38	0.04	0.02–0.08	<0.001	0.33	0.21–0.46	0.05	0.03–0.08	<0.001	0.0989	0.0947
13	0.15	0.08–0.27	0.03	0.01–0.05	<0.001	0.21	0.14–0.32	0.03	0.02–0.05	<0.001	0.0492	0.2010
14	0.13	0.07–0.23	0.01	0–0.02	<0.001	0.14	0.09–0.21	0.02	0.01–0.04	<0.001	0.4253	0.5667
15	0.09	0.04–0.14	0.01	0–0.01	<0.001	0.11	0.06–0.16	0.01	0–0.02	<0.001	0.2973	0.3831
16	0.06	0.04–0.11	0	0–0.01	<0.001	0.07	0.04–0.10	0	0–0.01	<0.001	0.9999	0.3718
17	0.04	0.02–0.09	0	0–0.01	<0.001	0.05	0.03–0.07	0	0–0.01	<0.001	0.6768	0.0058
18	0.03	0.01–0.07	0	0–0	<0.001	0.03	0.01–0.08	0	0–0	<0.001	0.1865	0.0944
19	0.02	0.01–0.05	0	0–0	<0.001	0.03	0.02–0.05	0	0–0	<0.001	0.2222	0.9173
20	0.02	0.01–0.04	0	0–0	<0.001	0.02	0–0.04	0	0–0	<0.001	0.9593	0.2243
21	0.01	0–0.03	0	0–0	<0.001	0.01	0–0.03	0	0–0	<0.001	0.7515	0.9219
22	0.01	0–0.03	0	0–0	<0.001	0	0–0.02	0	0–0	<0.001	0.7396	0.8423
23	0	0–0.2	0	0–0	<0.001	0.01	0–0.01	0	0–0	<0.001	0.2039	0.6025
24	0	0–0.01	0	0–0	<0.001	0	0–0.01	0	0–0	<0.001	0.6253	0.5100
25	0	0–0.01	0	0–0	<0.001	0	0–0.01	0	0–0	<0.001	0.4414	0.4696

^ stands for *p* value for women and men, * stands for *p* value between acceleration runs, # for deceleration runs respectively. Abbreviations: IQR—interquartile range.

**Table 4:** The comparison of the relative contributions of neutral runs (length 1–3) to the total number of sinus beats for all subjects (n = 101; men = 47 and women = 54) and the analysis of sex differences derived from 48 h ECG recordings of healthy individuals.

Run Length	All Participants n = 101 [%]		Women * n = 54 [%]		Men * n = 47 [%]		* *p*-Value (M-W)
	Median	IQR	Median	IQR	Median	IQR	
N1	0.50	0.39–0.69	0.57	0.43–0.73	0.48	0.38–0.67	0.08
N2	0.01	0.01–0.02	0.01	0.01–0.02	0.01	0.01–0.02	0.08
N3	0	0–0.001	0	0–0.001	0	0–0.001	0.84

*—comparison by the Mann–Whitney test. Abbreviations: IQR—interquartile range.

## Data Availability

The data presented in this study are available on request from the corresponding author. The data are not publicly available due to their sensitive nature.
